# Apela/Elabela/Toddler: New perspectives in molecular mechanism of heart failure

**DOI:** 10.21542/gcsp.2019.15

**Published:** 2019-09-20

**Authors:** Anthony P. Sunjaya, Angela F. Sunjaya, Frans Ferdinal

**Affiliations:** Department of Biochemistry and Molecular Biology, Faculty of Medicine, Tarumanagara University, Jl. Letjen S. Parman No. 1, Jakarta, Indonesia

## Abstract

**Background.** Despite significant therapeutic advances, heart failure (HF) remains unacceptably high in morbidity and mortality. Additionally, its high-care and costs make HF a deadly and costly disease. First reported independently by two group of researchers, Apela/Elabela/Toddler (ELA) is the second endogenous apelin-receptor ligand discovered which is encoded from a previously classified non-coding gene, and has emerged as a key signalling-pathway in the cardiovascular system.

**Aims.** To explore and summarise the biological effects and diagnostic potential of ELA as a new biomarker for heart failure.

**Results.** ELA (prepro-ELA 54 AA) is a molecule with three isoforms (ELA 11,16 and 32), recently identified as the second endogenous ligand to APJ-receptor and functions to mediate early cardiac development during zebrafish embryogenesis by inducing cardiogenesis, vasculogenesis and bone formation. In adults, it enhances cardiac contractility, promotes vasodilatory effects, mediates fluid homeostasis, reduces food intake, limits kidney dysfunction and exerts anti-atherosclerotic as well as anti-oxidative properties.

**Conclusion.** These results show that ELA, an endogenous agonist of the APJ-receptor exerts cardiovascular effects comparable and potentially more potent than apelin and is found to be downregulated in experimental models and humans with heart failure.

## Introduction

Heart failure is a complex clinical syndrome resulting in a reduced ability of the heart to pump and/or fill with blood, and is considered the fatal finishing line of all cardiovascular disorders. Despite significant therapeutic advances, heart failure (HF) remains unacceptably high in morbidity and mortality. Additionally, its high care requirements and associated costs make HF not only a deadly disease, but also a costly one. In 2014 an estimated 26 million people worldwide suffered with heart failure. In 2012 the United States reported over 10% of its total health expenditure (USD 31 billion) was spent on cardiovascular diseases with total costs expected to increase by 127% between 2012 and 2030^1,2^.

Significant efforts have been made in searching for new HF biomarkers^3^. First reported independently by two different group of researchers – Chng et al.^4^ and Pauli et al^5^, Apela/Elabela/Toddler (ELA) is the second endogenous apelin-receptor ligand discovered encoded from a previously classified non-coding gene and has emerged as a key signalling-pathway in the cardiovascular system.

### Apelin-APJ axis

Apelin is an endogenous hormone that binds to apelin receptors and exerts potent positive inotropic activity and increases coronary blood flow by vascular dilation, thereby providing beneficial effects in failing hearts^6^. The APJ, or apelin, receptor is a G-protein coupled receptor which shares similarity in homology to that of the angiotensin II type 1 receptor (54% homology in transmembrane domains and 32% homology for the entire sequence)^7,8^. Hence, the APJ system was found to negatively regulate angiotensin II signalling and upregulates angiotensin-converting enzyme 2 (ACE2) expression^9^. The Apelin-APJ axis was reported from mouse models to maintain heart contractility and cardioprotective effects under conditions of pressure overload, exercise and ageing^10,11^.

### Elabela/Toddler/Apela (ELA)

**Figure 1.**
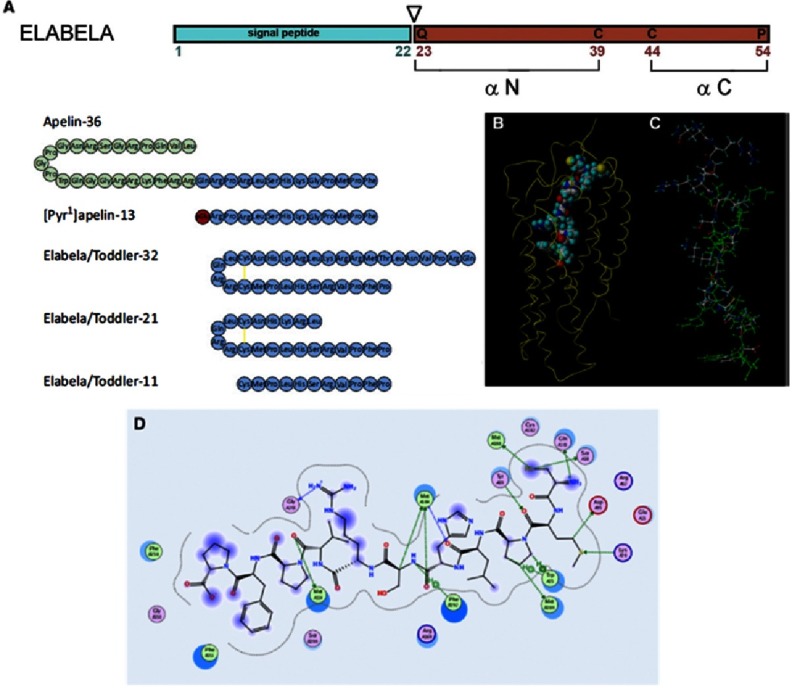
A – Sequence of apelin and Elabela/Toddler peptides^4,14^; B – Docking of ELA-11 on the apelin receptor; C – Apelin-13 (multicolored atoms) and ELA-11 (green) docking showing overlap in the binding site; D – Ligand interaction diagram showing key close contacts between ELA-11 and the apelin receptor model; ELA indicates Elabela/ Toddler/Apela^12^.

**Figure 2.**
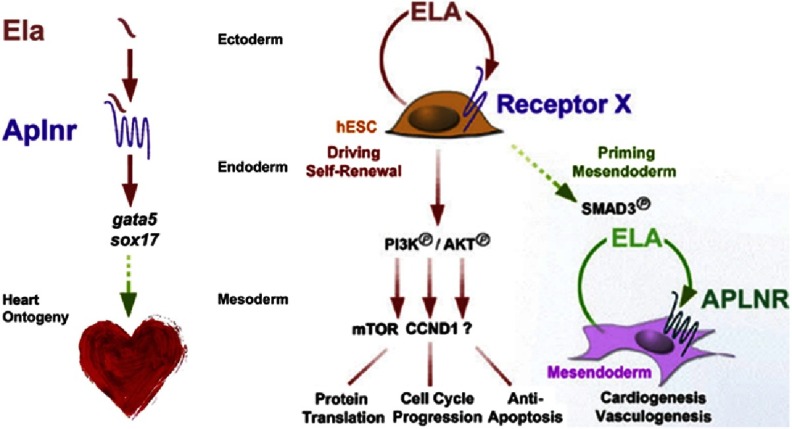
ELA’s effect during cardiogenesis^4,13^.

Earlier apelin has been thought to be the only ligand of the apela receptor, however recently ELA has been identified as the second endogenous ligand to this receptor. ELA (prepro-ELA 54 AA) is a molecule with 3 isoforms (ELA 11,16 and 32)^12^ and functions to mediate endoderm differentiation and cell migration hence promoting early cardiac development during zebrafish embryogenesis by inducing cardiogenesis, vasculogenesis and bone formation. Severe developmental defects including rudimentary or absent heart formations have been reported following ELA mutation similar to the effects of mutation of the Apela receptor^4,5^ (Figures 1 and 2).

Recent studies have also shown the potential effects of ELA in the self-renewal of human embryonic stem cells (hESC) in an autocrine manner which may be a result of its interaction with another unknown alternate receptor (receptor X)^13^ (Figure 2).

**Figure 3.**
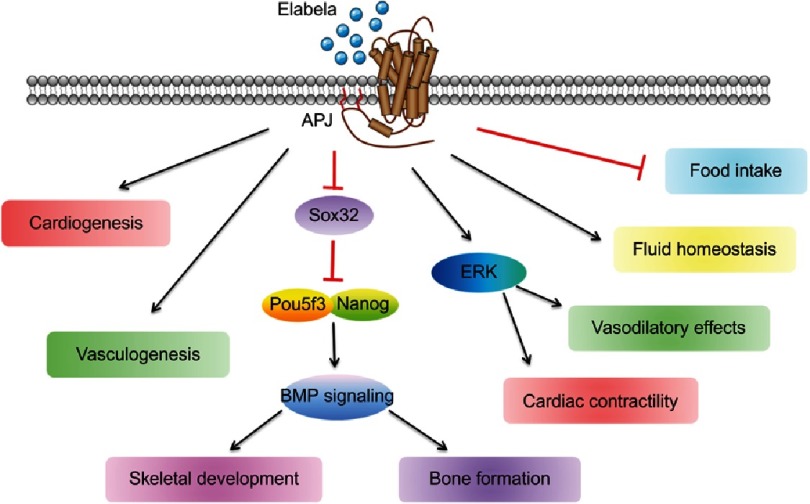
ELA’s effect during embryogenesis and in adults^16^.

In adults, ELA has been found localized in the endothelium and has an affinity for the human apelin receptor^4^. In addition, ELA has also been reported to be detected only in human stem cells (embryonic and induced adult pluripotent stem cells), and kidney unlike APJ receptors which have been reported to be found in heart, kidney, brain, lung, stomach, pancreas, fats and stem cells^13,15^. Hence, ELA was found to enhance cardiac contractility, promote vasodilatory effects, mediate fluid homeostasis, reduce food intake, limit kidney dysfunction and exerts anti-atherosclerotic as well as anti-oxidative properties^16^ (Figure 3).

With regards to fluid homeostasis, due to its greater distribution in the kidneys than apelin, ELA infusion resulted in a response 5 times that of apelin^17^. ELA treatment significantly suppressed the increased expression of mRNA associated with cardiac hypertrophy, such as brain natriuretic peptide (BNP), atrial natriuretic factor (ANF), and b-myosin heavy chain (b-Myhc)^7^, as well as markers of markers of myocardial injury such as lactate dehydrogenase (LDH), creatinine kinase-MB (CK-MB), Troponin I and markers of oxidative stress such as membrane lipid peroxidation (MDA), glutathione (GSH) and superoxide dismutase activities (SOD). Animal studies have also reported that cardiac function as measured by echocardiography improved significantly 2 weeks after ELA infusion post myocardial infarction^18^.

## Conclusion

These results show that ELA is an endogenous agonist of the APJ receptor exerting cardiovascular effects comparable and potentially more potent than apelin and downregulated in experimental models and humans with heart failure. ELA’s discovery open new possibilities in the field of biomarkers and therapeutics of heart failure due to its significance in the pathophysiology of heart failure. Further studies on ELA and APJ signalling are needed to provide more mechanistic insights into regulation and activation of APJ in cardiovascular diseases.

## Conflict of Interest

The authors declare no conflict of interest with regards to this publication.
